# The Effectiveness of Conservative Management for Acute Whiplash Associated Disorder (WAD) II: A Systematic Review and Meta-Analysis of Randomised Controlled Trials

**DOI:** 10.1371/journal.pone.0133415

**Published:** 2015-07-21

**Authors:** Taweewat Wiangkham, Joan Duda, Sayeed Haque, Mohammad Madi, Alison Rushton

**Affiliations:** 1 School of Sport, Exercise and Rehabilitation Sciences, University of Birmingham, Birmingham, B15 2TT, United Kingdom; 2 Primary Care Clinical Sciences, School of Health and Population Sciences, University of Birmingham, Birmingham, B15 2TT, United Kingdom; The James Cook University Hospital, UNITED KINGDOM

## Abstract

**Objective:**

To evaluate the effectiveness of conservative management (except drug therapy) for acute Whiplash Associated Disorder (WAD) II.

**Design:**

Systematic review and meta-analysis of Randomised Controlled Trials (RCTs) using a pre-defined protocol. Two independent reviewers searched information sources, decided eligibility of studies, and assessed risk of bias (RoB) of included trials. Data were extracted by one reviewer and checked by the other. A third reviewer mediated any disagreements throughout. Qualitative trial and RoB data were summarised descriptively. Quantitative syntheses were conducted across trials for comparable interventions, outcome measures and assessment points. Meta-analyses compared effect sizes with random effects, using STATA version 12.

**Data Sources:**

PEDro, Medline, Embase, AMED, CINAHL, PsycINFO, and Cochrane Library with manual searching in key journals, reference lists, British National Bibliography for Report Literature, Center for International Rehabilitation Research Information & Exchange, and National Technical Information Service were searched from inception to 15^th^ April 2015. Active researchers in the field were contacted to determine relevant studies.

**Eligibility Criteria for Selecting Studies:**

RCTs evaluating acute (<4 weeks) WADII, any conservative intervention, with outcome measures important to the International Classification of Function, Disability and Health.

**Results:**

Fifteen RCTs all assessed as high RoB (n=1676 participants) across 9 countries were included. Meta-analyses enabled 4 intervention comparisons: conservative versus standard/control, active versus passive, behavioural versus standard/control, and early versus late. Conservative intervention was more effective for pain reduction at 6 months (95%CI: -20.14 to -3.38) and 1-3 years (-25.44 to -3.19), and improvement in cervical mobility in the horizontal plane at <3 months (0.43 to 5.60) compared with standard/control intervention. Active intervention was effective for pain alleviation at 6 months (-17.19 to -3.23) and 1-3 years (-26.39 to -10.08) compared with passive intervention. Behavioural intervention was more effective than standard/control intervention for pain reduction at 6 months (-15.37 to -1.55), and improvement in cervical movement in the coronal (0.93 to 4.38) and horizontal planes at 3-6 months (0.43 to 5.46). For early (<4 days) versus late (>10 days) interventions, there were no statistically significant differences in all outcome measures between interventions at any time.

**Conclusions:**

Conservative and active interventions may be useful for pain reduction in patients with acute WADII. Additionally, cervical horizontal mobility could be improved by conservative intervention. The employment of a behavioural intervention (e.g. act-as-usual, education and self-care including regularly exercise) could have benefits for pain reduction and improvement in cervical movement in the coronal and horizontal planes. The evidence was evaluated as low/very low level according to the Grading of Recommendations Assessment, Development and Evaluation system.

## Introduction

Whiplash Associated Disorder (WAD) is a consequence of whiplash injury caused by rapid acceleration-deceleration of the head and neck, leading to bony and soft tissue injuries.[1] Road traffic accidents are the most common cause of whiplash.[2] Over the past 20 years, the incidence of traffic related whiplash has risen in most western countries.[3] Prevalence has been reported as 3/1,000 people in North America and Western Europe,[3] with 300,000 individuals experiencing WAD annually in the UK.[4] 40%- 60% of WAD patients develop chronicity [5–11] with approximately 30% of patients experiencing moderate to severe pain and disability.[12] Systematic reviews report limited effectiveness of chronic WAD management.[13–16] Consequently, effective intervention in the acute stage is required to prevent chronicity.

WAD contributes to a substantial economic burden throughout the industrialised world. Increased direct and indirect costs have been reported, including health care costs, reduced work productivity, lost earning capacity, higher socioeconomic costs and time contributed by caregivers.[17, 18] The annual economic cost related to WAD is estimated as $3.9 billion in the US [19] and €10 billion in Europe.[20] Insurance costs are also high in the western world,[3, 21–25] with the UK described as the ‘whiplash capital of Europe’ by the Association of British Insurers, who estimate that one person in 140 claims for whiplash injury annually.[24] In the UK, the cost of claims has risen from £7 to £14 billion over the past decade.[24]

Although there are five grades of whiplash classification, approximately 93% of patients post whiplash can be classified as WADII.[26] A neck complaint and musculoskeletal sign(s) are characteristic of WADII patients who are commonly managed by physiotherapists.[1] Conservative management (non-invasive treatment) is commonly utilized to manage acute WADII, and mainly focuses on physical treatment in terms of active exercise, manual techniques and physical therapy.[27, 28] Currently, the effectiveness of conservative interventions is still reported as limited in managing acute WADII.[29–38]

Patients with WAD exhibit both physical (e.g. pain and disability) and psychological (e.g. fear of movement, anxiety and depression) problems.[8, 25, 39–44] Currently, the psychological components (e.g. cognitive behavioural therapy and other behavioural approaches) of WADII management are under-explored, and this may be a factor contributing to the limited success of some approaches to management. Some clinical guidelines have suggested psychological strategies in managing chronic WAD II.[27, 28] However, these psychological components are not commonly recommended for management in the acute stage. Effectiveness of conservative management of acute WADII, employing both physical and psychological strategies is therefore important to prevent chronicity. No systematic review to date has specifically addressed this issue. The objective of this study was to evaluate the effectiveness of conservative management for acute WADII in order to summarise what we know about effective management in the acute stage.

## Materials and Methods

A systematic review of randomised controlled trials (RCTs) was conducted according to a pre-defined protocol using the method guidelines of the Back Review Group of the Cochrane Collaboration,[45] the Cochrane handbook,[46] and is reported in line with PRISMA.[47]

### Eligibility criteria

Table 1 details study eligibility criteria using Population Intervention Comparison Outcome Study Design (PICOS).[47]

**Table 1:** Eligibility criteria.

**Population**	Acute (<4 weeks) WADII (0-II or I-II participants were included when the grade was not classified in study)
**Intervention**	Conservative treatment (inclusive of the range of intervention detailed as part of the search strategy in search stages 3–20, and excluding drug therapy)
**Comparison**	Standard/control intervention
**Outcome**	Clinical relevant outcomes base on the International Classification of Function, Disability and Health (ICF)
**Study design**	Randomised controlled trial

### Information sources and searches

Two independent reviewers (TW/MM) searched:

- PEDro, Medline, Embase, AMED, CINAHL, PsycINFO, and Cochrane Library databases from inception to 15^th^ April 2015
- Key journals manually, including Spine, Manual Therapy, Physiotherapy, Physical Therapy, Australian Journal of Physiotherapy, Pain, and reference lists
- Dissertations and proceedings in the British National Bibliography for Report Literature, Center for International Rehabilitation Research Information & Exchange, Index to Scientific and Technical Proceedings, National Technical Information Service, and System for Information on Grey Literature

Finally, active researchers in the field were contacted to identify other relevant studies.

#### Examples of Search Strategies. Medline (Ovid) 1946 – 15^th^ April 2015 and Embase 1974 – 15^th^ April 2015

1. Acute whiplash OR acute whiplash injury* OR acute whiplash associated disorder* OR acute WAD OR acute whiplash associated disorder* II OR acute WAD II OR whiplash associated disorder* OR WAD OR whiplash associated disorder* II OR WAD II, OR whiplash OR whiplash injury* OR whiplash patient* OR whiplash syndrome* OR cervical spine disorder* OR cervical spine injury*
2. Randomized controlled trial* OR randomised controlled trial* OR randomized clinical trial* OR randomised clinical trial* OR randomized controlled clinical trial* OR randomised controlled clinical trial* OR RCT*
3. Conservative treatment* OR conservative intervention* OR conservative management* OR conservative approach OR conservative therapy*
4. 1 AND 2
5. 3 AND 4
6. Physiotherapy OR physical therapy OR physical approach OR physical intervention OR physical management
7. 4 AND 6
8. Manual therapy OR manipulation OR mobilization OR massage
9. 4 AND 8
10. Exercise OR exercise therapy OR active intervention* OR active treatment* OR active exercise OR range of motion exercise OR ROM exercise OR strengthening exercise OR stretching exercise OR therapeutic exercise OR endurance exercise OR endurance training OR home exercise OR proprioception exercise
11. 4 AND 10
12. Electrotherapy OR electrical stimulation OR transcutaneous electrical nerve stimulation OR TENS OR percutaneous electrical nerve stimulation OR PENS OR frequency-modulated electromagnetic neural stimulation OR FREMS OR electromagnetic therapy OR electromagnetic field OR electromagnetic field therapy OR pulse electromagnetic field OR PEMF OR pulse magnetic field OR static magnetic field OR electrical spinal cord stimulation OR SCS OR microcurrent transcutaneous electrical nerve stimulation OR micro-TENS OR high-frequency external muscle stimulation OR external muscle stimulation OR HF OR interferential current OR IFC OR Russian current OR faradic current OR intermittent direct current OR IDC OR galvanic current OR GC OR direct current OR DC OR diadynamic current OR high voltage galvanic current OR HVGC OR microcurrent electrical nerve stimulation OR MENS OR electroacupuncture
13. 4 AND 12
14. Thermotherapy OR heat OR hot pack ultraviolet OR UV OR infrared radiation OR IR OR infrared therapy OR laser OR laser therapy OR ice OR cold therapy OR ice massage OR ice pack OR contrast bath OR cryotherapy
15. 4 AND 14
16. Posture OR balance OR traction
17. 4 AND 16
18. Education OR educational intervention OR patient education OR self-management OR self-management program OR neck school OR whiplash school
19. 4 AND 18
20. Behaviour approach* OR behaviour therapy* OR behaviour treatment* OR cognitive behaviour OR cognitive therapy* OR cognitive treatment* OR cognitive behaviour approach* OR cognitive behaviour therapy* OR psychological approach* OR psychological aspect*
21. 4 AND 20

### Study selection

After searching, the two independent researchers (TW/MM) evaluated identified studies for eligibility by screening 1] title and abstract, then 2] full texts, grading each study as eligible/ not eligible/ might be eligible at each stage.[45] Included studies were agreed by the two reviewers (TW/MM). The third reviewer (AR) mediated in situations of disagreement. Only full articles in English were included.

### Data collection process

Data were extracted by one researcher (TW) and checked by a second (MM). Trial authors were contacted for additional data when data were missing or ambiguous.

### Data items

Trial authors, countries, study design, stage of whiplash patients, WAD classification, sample size, interventions, study setting, power calculations, outcome measures, follow-up period, loss of follow-up, intention to treat and main results were extracted for each trial. Data relating to key outcome measures including pain, disability, function, patient satisfaction, social impact, and physical impairment based on the International Classification of Functioning, Disability and Health were extracted.[48]

### Risk of bias (RoB) in individual trials

Training and a pilot of RoB assessment was carried out by the two reviewers (TW/MM). The two reviewers evaluated RoB for each included trial independently. The Cochrane RoB assessment tool, which is informed by empirical evidence, was utilised to assess the internal validity / risk of bias.[47, 49, 50] The third reviewer (AR) mediated situations of disagreement following discussion. Each component of RoB was reported in terms of unclear, low or high risk of bias in tabular form.[49, 50] The Kappa Measure of Agreement was utilised to assess the agreement between the two reviewers using SPSS version 21.

### Summary measures

Risk of bias was assessed using the Cochrane RoB assessment tool.[49, 50] The recommendation for overall RoB was in line with the Cochrane handbook.[46, 49, 50] Quantitative data analysis was conducted in situations of comparability of interventions, outcome measures and assessment points across trails. Meta-analyses compared effect sizes with random effects as the primary analyses and were conducted using STATA software version 12.[51]

### Synthesis of results

Standardised mean difference (SMD) and standard error of SMD were calculated for meta-analyses. The results of meta-analyses were graphically demonstrated in forest plots. Summary statistics including 95% Confident Interval (CI), p-value, and heterogeneity (I^2^) were tabulated.

### Risk of bias across studies

RoB assessment across studies was tabulated. The criteria of judgement for overall RoB followed recommendations in the Cochrane Handbook.[50] A consensus in overall potential risk of bias was explored to evaluate the level of evidence. As the number of studies in each meta-analysis was less than 10, evaluation of publication bias using Funnel plots was not helpful.[52, 53]

## Results

### Study selection

**Fig 1:**
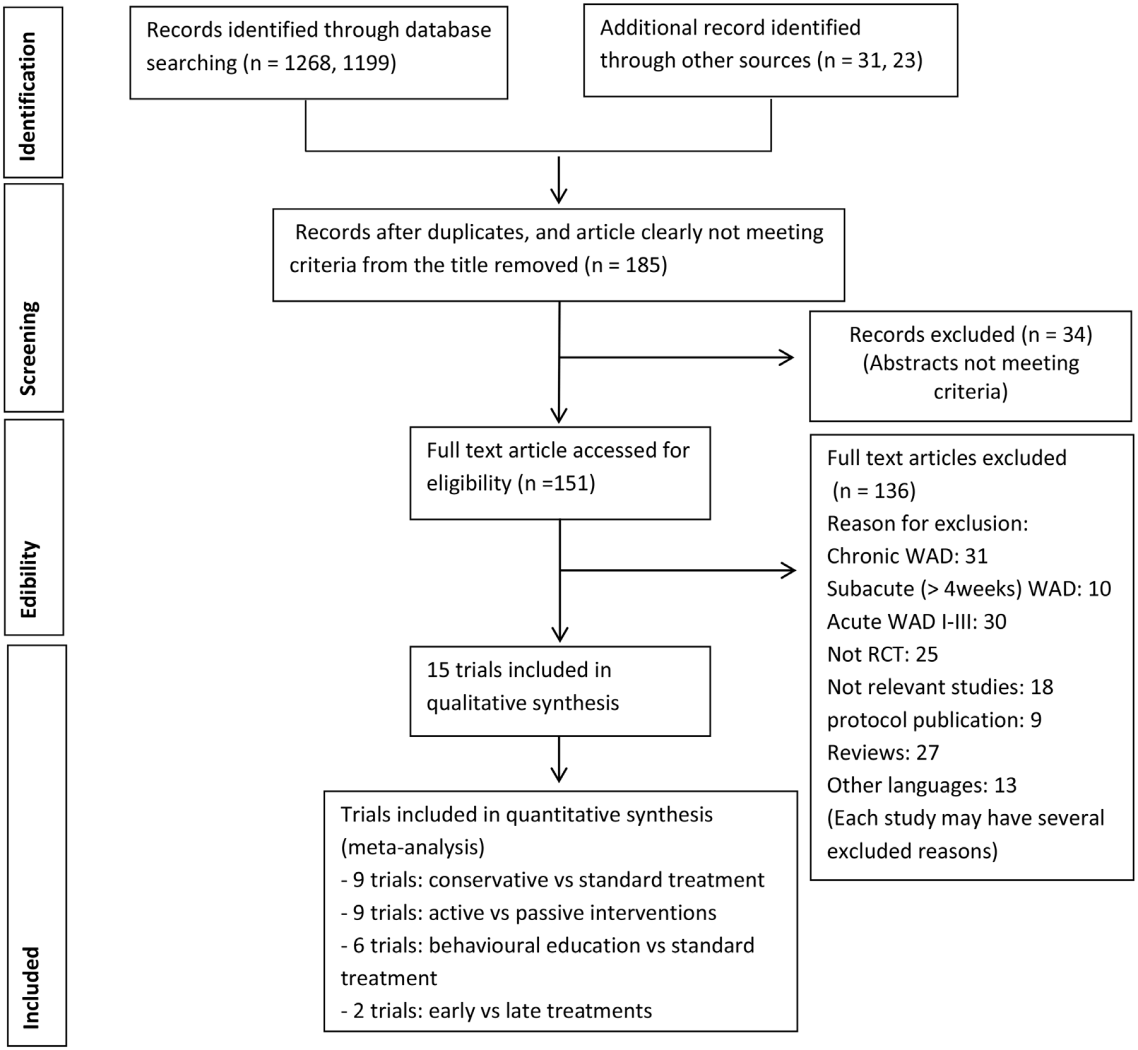
Study selection flow diagram.

15 RCTs (n = 1676 participants, 9 countries) were included. The process of study selection is detailed in Fig 1.

### Study characteristics

Trial characteristics are summarised in Table 2. A range of conservative treatments were employed across included trials (see Table 2 for detail of interventions). Interventions could be grouped to inform analyses into conservative (broad group), active, passive and behavioural interventions.

**Table 2:** Summary results from the 15 included trials.

Studies	Countries	N	WAD	Design	Intervention 1	Intervention 2	Intervention 3	Outcome Measures	Follow-up period	Main Results
Aigner et al. 2006 [29]	Austria	53	II	Parallel RCT with single blind	Collar and laser acupuncture	Collar and placebo laser acupuncture	-	-CROM,Subjective symptoms (neck pain, dizziness, paresthesia and tinnitus),Sick leave	3 weeks (Clinically)8-12 Months (Postal)	No significant difference between interventions in all outcome measures
Bonk et al. 2000 [30]	Germany	147	0-II	Parallel RCT	Active therapy (active mobilization and exercise)	Collar therapy	Control	-Subjective symptoms (such as pain, stiffness), CROM	3 months	No significant difference between interventions at 3 months
Borchgrev-ink et al. 1998 [31]	Norway	201	0-II	Parallel RCT with single blind	Act-as-usual	Immobilisati-on	-	-Subjective symptoms using questionnaire, Pain (VAS), CROM, Shoulder movement, Sick leave	6 months	I1>I2 significantly in improvement neck pain (p<0.01), pain during daily activities (p< 0.05), headaches (p<0.01), painful regions (p<0.01), and memory and concentration problems (p<0.001) at 6 months. ROM of neck and shoulder did not differ between interventions.
Conforti and Fachinetti 2013 [54]	Italy	135	I-II	Parallel RCT with single blind	HPLT	PT (manual therapy, passive and active exercise)	-	-Pain (VAS), The date of return to work	6 weeks	I1 > I2significantly improved in both pain (p = 0.005) and the date of return to work (p<0.001)
Dehner et al. 2006 [55]	Germany	70	II	Parallel RCT	2 days with collar + standard PT after a weeks	10 days with collar + standard PT after a weeks	-	-Pain (VAS), Disability (VAS), CROM	6 months	No significant difference between interventions in all outcome measures
Dehner et al. 2009 [32]	Germany	90	II	Parallel RCT	Active physical therapy	Passive physical therapy	Act as usual	-Pain (VAS), CROM, Period of disability/ sickness costs	2 months	- Pain improvement: I1>I2>I3 significantly- CROM: I1 = I2 = I3- Period of disability: I1 = I2<I3
Ferrari et al. 2005 [33]	Canada	112	I-II	Parallel RCT with single blind	Education pamphlet	Control group	-	-The number of recovery	3 months	No significant difference between interventions
Foley-Nolan et al. 1992 [34]	Ireland	40	0-II	Parallel RCT with single blind	PEMT + collar + active exercise	Placebo + collar + active exercise	-	-Pain (VAS), CROM, Number of analgesics	3 months	I1>I 2 significantly improved in terms of pain (VAS) at 2 and 4 weeks but no significance at 12 weeks. For the CROM, I1>I2 significantly at 3 months (p<0.001).
Jull et al. 2013 [56]	Australia	101	II	Parallel RCT with single blind	Multiprofess-ional intervention	Usual care	-	-Pain (VAS), NDI, IES, PFActS-C, GHQ 28, CROM, Craniocervical flexor test, Balance, Cervical proprioception, PPT, HPT, CPT, Sympathetic vasoconstrictor response, Types and dosage of medications	12 months	No significant difference between interventions in all outcome measures but the recovery of pain and disability between baseline, 6 and 12 months has significant differences in both interventions.
Ottosson et al. 2007 [35]	Sweden	127	I-II	Parallel RCT with unblind	Educational programme + self-care (exercise for relaxation and postural control)	Standard Rx.(basic medications)	-	-Self-reported recovery, SF-36, SMFA, Pain and mental distress (VAS), Sick leave	12 months	I1>I2 significantly in terms of self-reported recovery (p<0.03) but no significant difference in other outcomes between interventions
Picelli et al. 2011 [36]	Italy	18	I-II	Parallel RCT with single blind	Neck fascia manipulation	Neck mobilization exercise + stretching	-	-CROM, Pain (VAS), NDI, PPT	2 weeks	CROM: I1>I2 significantly (p<0.01) but other outcome measures, no significant differences between interventions.
Rosenfeld et al. 2003 [37][Rosenfeld et al. 2006 reporting same trail][57]	Sweden	102	0-II	Parallel RCT with single blind	Active mobilization within 96 hours or Active mobilization 14 days	Standard Rx. (rest, collar and gradual self-mobil)within 96 hrs or Standard Rx. (rest, collar and gradual self-mobil) 14 days	-	-Pain (VAS), CROM, Sick leave	3 years	Pain and sick leave I1<I2 significantly (p<0.05) but no significance in CROM (p = 0.06–0.08)
Schnabel et al. 2004 [58]	Germany	200	I-II	Parallel RCT with un- blind	Mobilisation exercise	Collar therapy	-	-Pain (VAS), Disability (VAS)	6 weeks	I1>I2 significantly in improving pain and disability (p<0.05)
Scholten-Peerters et al. 2006 [59]	Netherlands	80	I-II	Parallel RCT with single blind	Education and exercise by PTs	Education by GPs	-	-Pain (VAS), Functional recovery (VAS), SF-36, CROM, TSK, PCI, NDI, Disability in housekeeping and social activities (VAS)	13 months	At 12 weeks, I1>I2 significantly for CROM improvement but in long term I2>I1 significantly in terms of functional recovery, coping, and physical functioning.
Vassiliou et al. 2006 [38]	Germany	200	I-II	Parallel RCT with unblind	PT + active exercise	Standard Rx (drugs + soft collar)	-	-Pain and disability (NRS), Number of days with oral medication, The period of soft collar	6 months	I1>I2 significantly improved in terms of pain intensity and disability. Other outcomes had been reported but no compare using statistic procedure.

CPT: Cold Pain Threshold, CROM: Cervical Range of Motion, GHQ 28: General Health Questionnaire, HPLT: High Power Laser Therapy, HPT: Hot Pain Threshold, I: Intervention, IES: Impact of Events Scale, NDI: Neck Disability Index, NRS: Numeric Rating Scale, PCI: Pain Coping Inventory, PFActS-C: Pictorial Fear of Activity Scale-Cervical, PPT: Pressure Pain Thresholds, PT: Physiotherapy, RCT: Randomised Controlled Trail, Rx: Treatment, SMFA: Short Musculoskeletal Function Assessment, SF-36: Functional Health Status (Short Form 36), TSK: Tampa Scale for Kinesiophobia, VAS: Visual Analogue Scale

### Risk of bias within and across trials

Agreement of RoB assessment between two reviewers was very good (Kappa 0.87).[60] The RoB of individual trials is detailed in Table 3. All trials were assessed as high RoB for a range of reasons including: inadequate allocation concealment, selective outcome reporting, no intention to treat analysis, no specification of primary outcome, no specification primary endpoint, no reporting statistical analysis, no reporting reasons of drop-out, difference in loss to follow up between groups, and no reporting of some outcome measures or/and information.

**Table 3:** Summary of risk of bias assessment [61].

Studies	Components of risk of bias							Summary risk of bias	Overall RoB	Comments, high risk components
	1	2	3	4	5a	5b	6			
Aigner et al. 2006 [29]	U	U	U	L	H	H	H	High (3) Unclear (3) Low (1)	H	Three high component: 5a, 5b, 6 (5a: No primary outcomes pre-specified, 5b: No primary outcomes pre-specified, 6: No primary endpoint specified and No ITT reported)
Bonk et al. 2000 [30]	U	U	H	L	H	N/A	H	High (3) Unclear (2) Low (1) N/A (1)	H	Three high component: 3, 5a, 6 (3: Unblind assessors, 5a: No primary outcomes pre-specified, 6: No primary endpoint specified and No ITT reported)
Borchgrevink et al. 1998 [31]	U	U	L	H	U	N/A	H	High (2) Unclear (3) Low (1) N/A (1)	H	Two high component: 4, 6 (4: loss follow-up >16 without stating of which group, 6: No primary endpoint specified and No ITT reported)
Conforti and Fachinetti 2013 [54]	U	U	L	L	H	N/A	H	High (2) Unclear (2) Low (2) N/A (1)	H	Two high component: 5a, 6 (5a: No primary outcomes pre-specified, 6: No primary endpoint specified, No ITT reported and No statistical analysis reported)
Dehner et al. 2006 [55]	L	L	U	L	H	N/A	H	High (2) Unclear (1) Low (3) N/A (1)	H	Two high component: 5a, 6 (5a: No primary outcomes pre-specified, 6: No primary endpoint specified and No ITT reported)
Dehner et al. 2009 [32]	L	L	U	U	H	N/A	H	High (2) Unclear (2) Low (2) N/A (1)	H	Two high component: 5a, 6 (5a: No primary outcomes pre-specified, 6: No primary endpoint specified and No ITT reported)
Ferrari et al. 2005 [33]	L	L	L	H	L	N/A	H	High (2) Unclear (1) Low (4) N/A (1)	H	Two high component: 4, 6 (4: reasons of drop-out were not reported, 6: No primary endpoint specified and No ITT reported)
Foley-Nolan et al. 1992 [34]	U	U	U	L	H	N/A	H	High (2) Unclear (3) Low (1) N/A (1)	H	Two high component: 5a, 6 (5a: No primary outcomes pre-specified, 6: No primary endpoint specified and No ITT reported)
Jull et al. 2013 [56]	L	L	L	U	L	L	H	High (1) Unclear (1) Low (5)	H	One high component: 6 (No ITT and Pain (VAS) has reported in significant difference between group at baseline)
Ottosson et al. 2007 [35]	L	L	H	L	N/A	L	H	High (2) Low (4) N/A (1)	H	Two high component: 3, 6 (3: Unblind, 6: No primary endpoint specified)
Picelli et al. 2011 [36]	L	U	L	L	H	N/A	H	High (2) Unclear (2) Low (3) N/A (1)	H	Two high component: 5a, 6 (5a: P-value did not report in NDI and PPT, 6: No primary endpoint specified and No ITT reported)
Rosenfeld et al. 2003 [37][Rosenfeld et al. 2006 reporting same trail][57]	L	L	L	H	H	H	H	High (4) Low (3)	H	Four high component: 4, 5a, 5b, 6 (4: drop-out difference between groups, 5a: No primary outcomes pre-specified, 5b: No primary outcomes pre-specified, Reporting sick leave but have not stated, 6: No primary endpoint specified and No ITT reported)
Schnabel et al. 2004 [58]	U	U	U	H	H	N/A	H	High (3) Unclear (3) N/A (1)	H	Three high component: 4, 5a, 6 (4: Loss of follow-up: A = 36%, B = 15%, 5a: No primary outcomes pre-specified, 6: No primary endpoint specified and No ITT reported)
Scholten-Peeters et al. 2006 [59][Scholten-Peeters et al. 2003 trial protocol][62]	L	L	L	L	L	L	H	High (1) Low (6)	H	One high component: 6 (No primary endpoint specified)
Vassiliou et al. 2006 [38]	L	L	L	L	L	N/A	H	High (1) Low (4) N/A (5)	H	One high component: 6(No primary endpoint specified)

1 = Sequence generation, 2 = Allocation concealment, 3 = Blinding of participants, personnel and assessors, 4 = Incomplete outcome data, 5a = Selecting outcome reporting (Short term), 5b = Selecting outcome reporting (Long term), 6 = Other potential threats to validity, L = low risk of bias, H = high risk of bias, U = unclear risk of bias, N/A = not applicable

### Results of individual trials

Based on the comparability of interventions, outcome measures and assessment points across trials, meta-analyses were conducted on 4 intervention comparisons (please see Table 2 for details of specific interventions): conservative [any non-invasive intervention] versus standard/control (9 RCTs, n = 1182 participants),[29–31, 33–35, 37, 38, 58] active [activities from health professional suggestion to improve symptoms or reduce suffering from illness] versus passive [any intervention which use other people, equipment or other things to reduce symptoms or illness] (9 RCTs, n = 1145 participants),[30–32, 35–38, 58, 59] behavioural [strategies to promote useful behaviour to improve symptoms or reduce illness] versus standard/control (6 RCTs, n = 987 participants),[30, 31, 33, 35, 38, 58] and early [< 1 week] versus late [>2 week] (2 RCTs, n = 172 participants).[37, 55] A summary of the meta-analyses is detailed in Table 4.

**Table 4:** Summary meta-analyses.

Meta-analyses	I^2^ (%)	95% Confidence Interval (CI)	p-value
**Conservative vs standard/control interventions**			
Pain intensity (VAS)			
at <3 months	70.0	-12.90, 2.19	0.164
at 6 months	63.8	-20.14, -3.38	0.005*
at 1–3 years	0.0	-25.44, -3.19	0.012*
CROM in sagittal plane			
at 6 months	10.9	-3.61, 9.17	0.394
at 3 years	60.5	-7.78, 27.69	0.271
CROM in coronal plane			
at <3 months	64.2	-5.78, 6.48	0.911
at 6 months	0.0	-1.89, 6.42	0.285
at 3 years	64.1	-5.83, 16.99	0.338
CROM in horizontal plane			
at < 3 months	0.0	0.43, 5.60	0.022*
at 6 months	55.1	-16.04, 8.48	0.545
at 3 years	19.0	-6.80, 16.51	0.415
Sick leave (days)	0.0	-39.02, 3.83	0.107
**Active vs passive interventions**			
Pain intensity (VAS)			
at <3 months	76.3	-10.51, 6.07	0.599
at 6 months	56.9	-17.19, -3.23	0.004*
at 1–3 years	0.0	-26.39, -10.08	< 0.001*
CROM in sagittal plane			
at <3 months	80.6	-17.73, 8.69	0.452
at 6 months	0.0	-1.69, 8.44	0.192
at 3 years	60.5	-7.78, 27.69	0.271
CROM in coronal plane			
at <3 months	82.2	-6.94, 6.15	0.905
at 6 months	0.0	-1.13, 5.99	0.180
at 3 years	64.1	-6.83, 16.99	0.338
CROM in horizontal plane			
at <3 months	85.2	-8.96, 12.35	0.755
at 6 months	69.4	-11.28, 12.81	0.892
at 3 years	19.0	-6.80, 16.51	0.415
Sick leave (days)	0.0	-39.02, 3.83	0.107
**Behavioural vs standard/control interventions**			
Pain intensity (VAS)			
at 6 weeks	70.0	-12.90, 2.19	0.164
at 6 months	44.2	-15.37, -1.55	0.016*
CROM in coronal plane			
at 3–6 months	0.0	0.93, 4.38	0.003*
CROM in horizontal plane			
at 3–6 months	0.0	0.43, 5.46	0.027*
**Early vs late interventions**			
Pain intensity (VAS)			
at 6 months	79.2	-25.74, 18.21	0.737
at 3 years	0.0	-12.51, 9.85	0.816
CROM in sagittal plane			
at 6 months	51.3	-13.16, 17.02	0.802
at 3 years	60.5	-11.35, 24.04	0.482
CROM in coronal plane			
at 6 months	27.0	-8.93, 6.92	0.803
at 3 years	64.0	-12.95, 9.96	0.799
CROM in horizontal plane			
at 6 months	75.9	-20.66, 27.31	0.786
at 3 years	19.1	-4.86, 20.25	0.230
Sick leave (days)	0.0	-13.37, 28.40	0.481

* Statistical significance (p < 0.05)

### Synthesis of results

**Fig 2:**
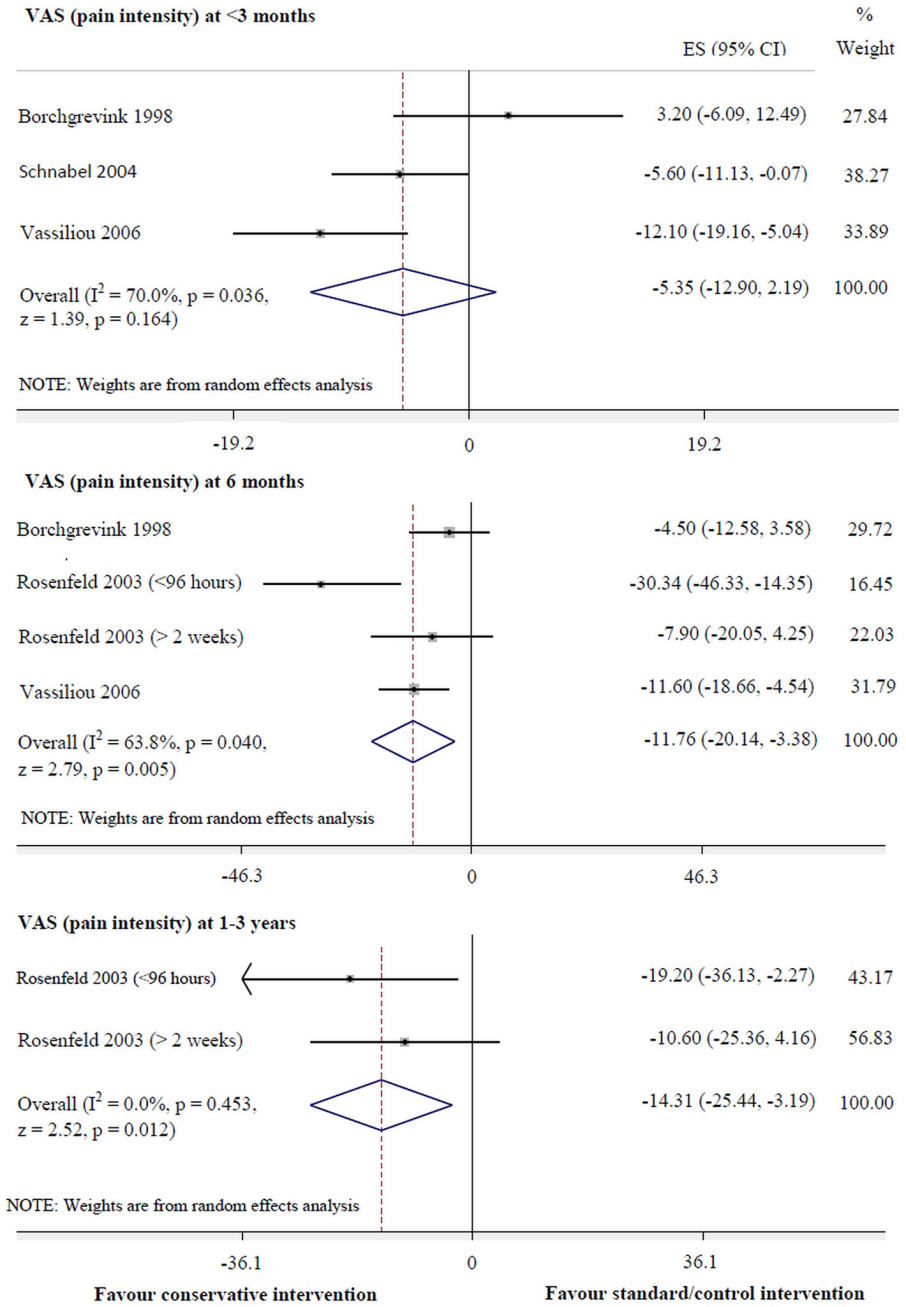
Conservative versus standard/control interventions for VAS (pain intensity).

**Fig 3:**
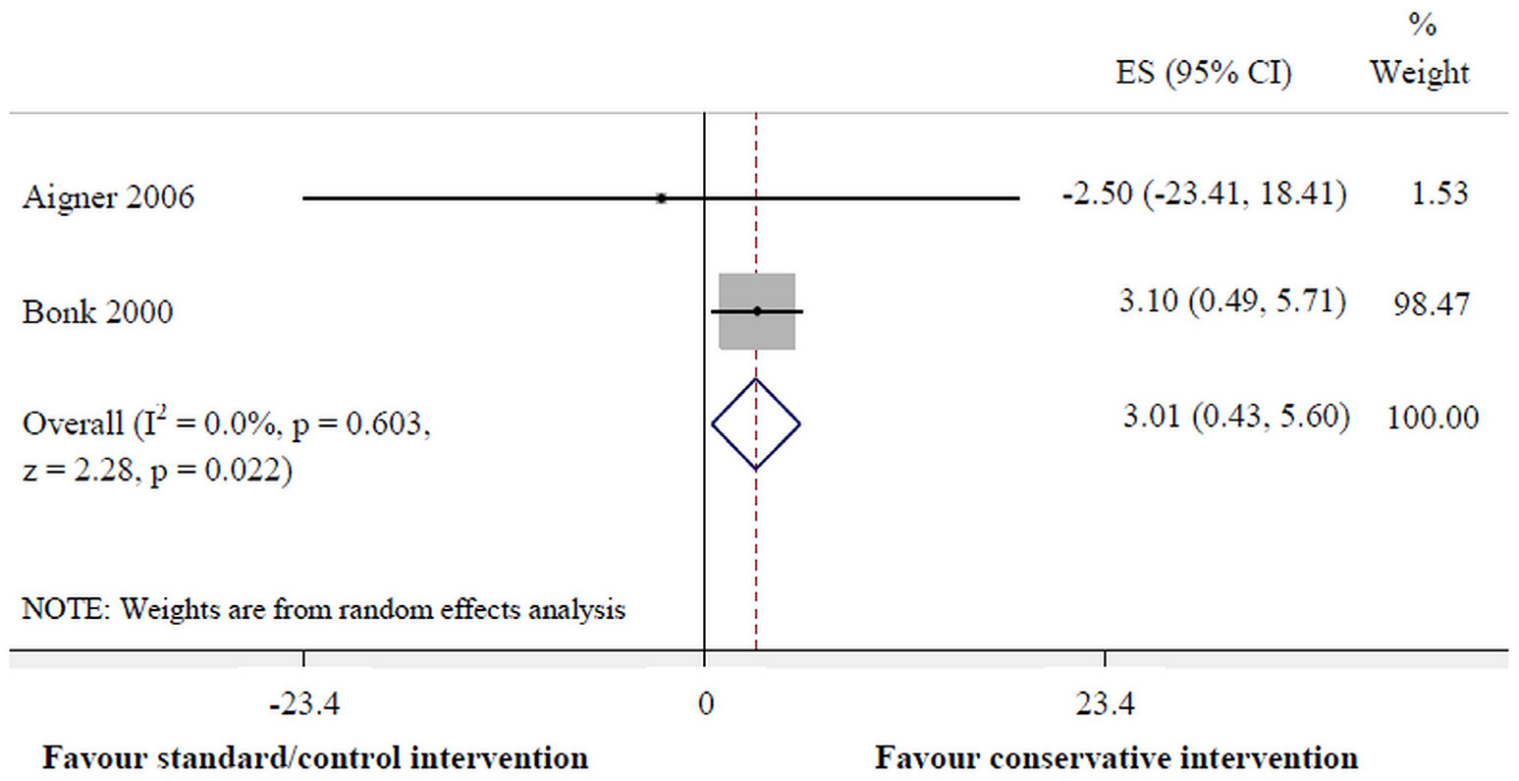
Conservative versus standard/control interventions for cervical horizontal movement at <3 months.

Conservative intervention was more effective for pain reduction than standard/control intervention at 6 months (95% CI: -20.14 to -3.38, p = 0.005, I^2^ = 63.8%) and 1–3 years (-25.44 to -3.19, p = 0.012, I^2^ = 0.0%) (Fig 2). Moreover, conservative intervention was superior to the standard/control intervention for improvement of cervical mobility in the horizontal plane at <3months (0.43 to 5.60, p = 0.022, I^2^ = 0.0%) (Fig 3). However, there were no significant differences between interventions for pain reduction at <3months, other follow-up periods in the horizontal plane of Cervical Range of Motion (CROM), nor any follow-up periods in terms of other planes of CROM, including the number of days of sick leave.

**Fig 4:**
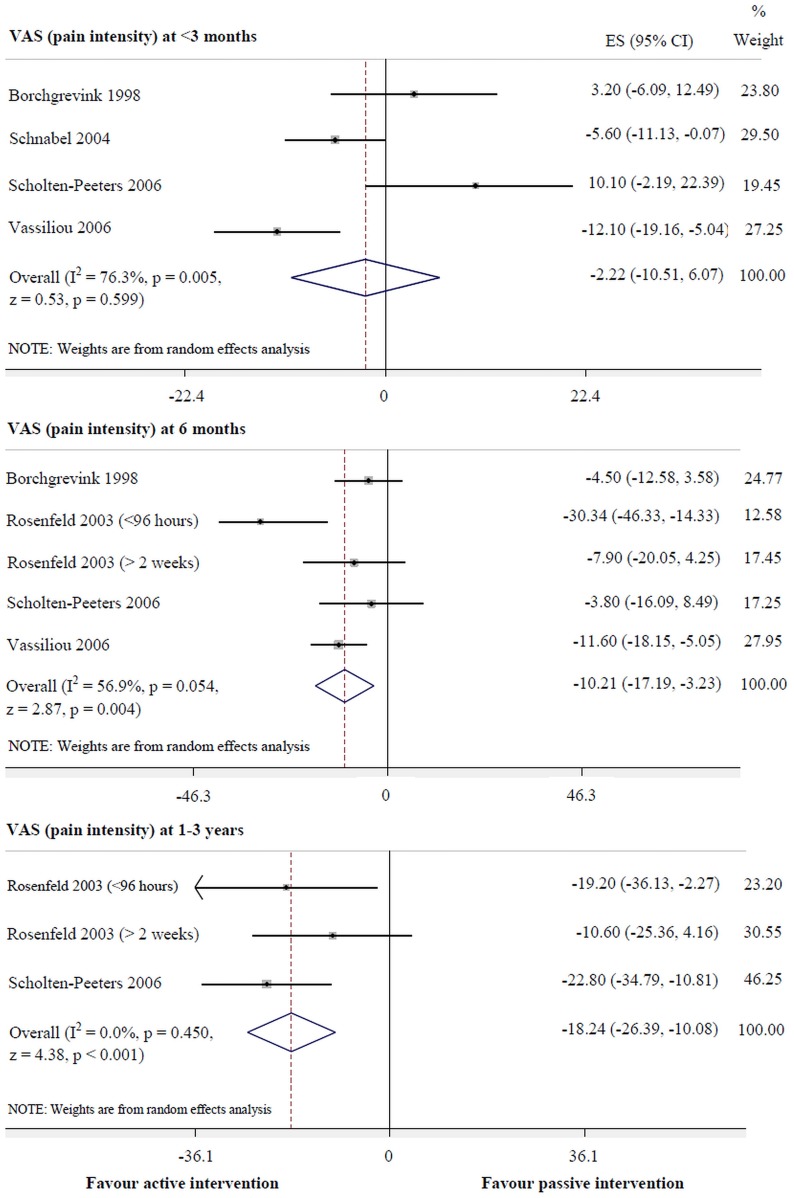
Active versus passive interventions for VAS (pain intensity).

Active intervention was more effective than passive intervention for pain reduction at 6 months (-17.19 to -3.23, p = 0.004, I^2^ = 56.9%) and 1–3 years (-26.39 to -10.08, p<0.001, I^2^ = 0.0%) (Fig 4). However, there was no significant difference in pain reduction at <3 months. Also, improvement of cervical mobility and days of sick leave were not significantly different between interventions.

**Fig 5:**
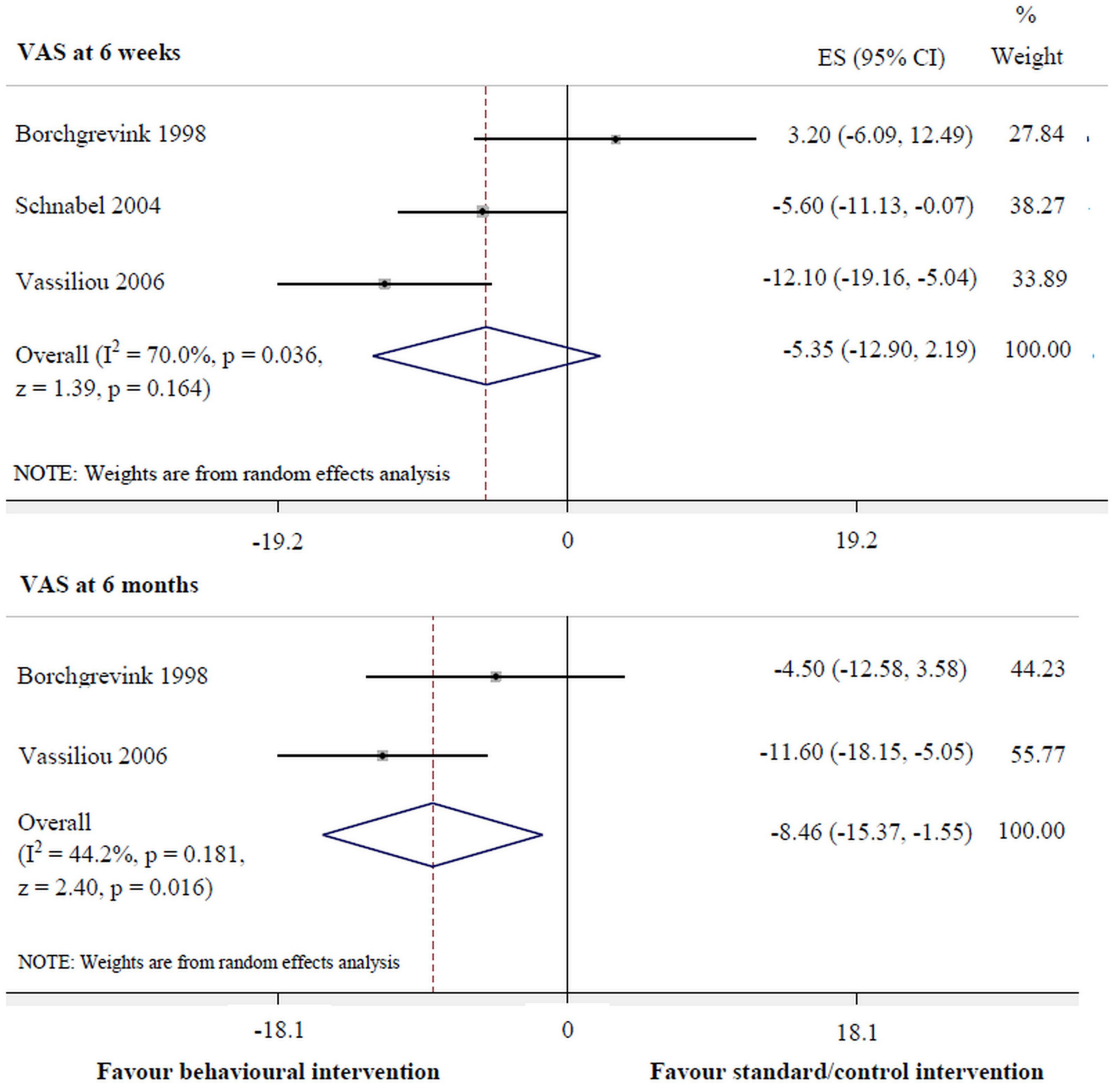
Behavioural versus standard/control interventions for VAS (pain intensity).

**Fig 6:**
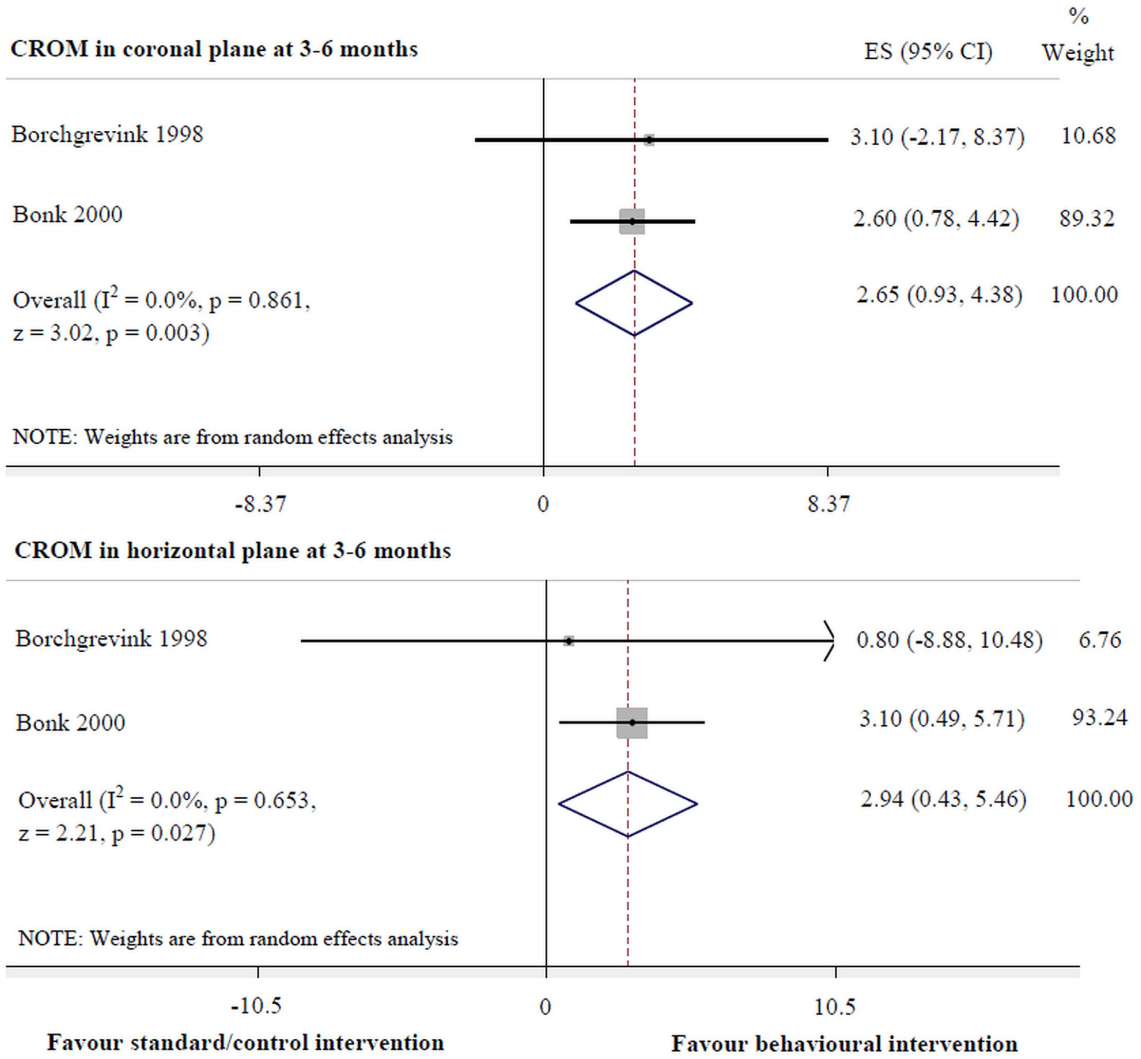
Behavioural versus standard/control interventions for cervical movement.

Behavioural intervention was more effective for pain reduction at 6 months (-15.37 to -1.55, p = 0.016, I^2^ = 44.2%) (Fig 5) and improvement of cervical mobility in the coronal (0.93 to 4.38, p = 0.003, I^2^ = 0.0%) and horizontal planes (0.43 to 5.46, p = 0.027, I^2^ = 0.0%) at 3–6 months, compared with the standard/control intervention (Fig 6). However, there was no significant difference between interventions for pain reduction at 6 weeks.

There were no significant differences between early and late interventions for pain reduction, CROM, and days of sick leave at any follow-up period.

## Discussion

### Summary of evidence

15 RCTs with high RoB were included. Some trials [30, 31, 34] may be high risk of bias owing to poor reporting as published prior to the CONSORT reporting guidelines.[63, 64] Although trial reporting in terms of primary outcome, sample calculation, random sequence generation and allocation concealment significantly improved between 2000 and 2006,[65] the quality of reporting blinding, and descriptions of approach, exclusion, treatment and missing data is still frequently inadequate,[65–67] contributing in 2010 to the revised CONSORT statement.[68, 69] In this systematic review, only three trials were published after 2010.[36, 54, 56] Due to the high RoB across all trials, confidence in findings is reduced.

The meta-analyses findings are more powerful and reliable than individual trials because of minimised biases from the individual trials.[70] The results of the meta-analyses were influenced by individual trials demonstrating conflicting conclusions. For example, the meta-analysis demonstrated that conservative intervention was more effective than standard/control intervention for pain reduction long term, despite some trials reporting negative finding.[35, 37] Another example is that some trials [32, 38, 58] found the active intervention was more effective than the passive short term, but there was no effect demonstrated in the meta-analysis.

The level of heterogeneity was evaluated to determine the credibility of the evidence.[71] For example, in the meta-analyses demonstrating an effect for pain reduction at 6 months, the heterogeneity ranged from moderate (I^2^ = 44.2%, behavioural intervention) to substantial (I^2^ = 63.8%, conservative intervention; I^2^ = 56.9%, active intervention), and this was acceptable overall.

Although this systematic review identified some interventions demonstrating an effect, the size of the effect was not clinically significant. The minimal clinical significant differences in improvement of pain intensity (VAS) and CROM are at least 20 millimetres [72] and 10°,[73] respectively. Currently, therefore, there is no evidence of an effective intervention for acute WADII management. However, conservative intervention (non-invasive treatment inclusive of both physical and psychological components such as active mobilisation exercise, manual techniques, physical agents, multimodal therapy, behavioural approaches, and education, except for drug therapy) seems to be a useful intervention for acute WADII management in terms of pain reduction in the medium (95% CI: -20.14 to -3.38, p = 0.005, I^2^ = 63.8%) to long term (95%CI: -25.44 to -3.19, p = 0.012, I^2^ = 0.0%), and improvement of cervical mobility in the horizontal plane in the short term (95%CI: 0.43 to 5.60, p = 0.022, I^2^ = 0.0%) compared with standard/control intervention.

From these findings, there are two interesting interventions for acute WADII management worthy of further consideration. Firstly, active intervention (involving range of movement, mobilising exercises, and strengthening of the neck and scapular muscles) is strong recommended from whiplash guidelines [27, 28] and may be useful for pain reduction medium (95%CI: -17.19 to -3.23, p = 0.004) to long term (95%CI: -26.39 to -10.08, p = <0.001). Secondly, behavioural intervention (e.g. act-as-usual, education and self-care including regularly exercise) may be effective for pain reduction medium term (95%CI: -15.37 to -1.55, p = 0.016) and improvement of cervical mobility in the coronal (95%CI: 0.93 to 4.38, p = 0.003) and horizontal planes (95%CI: 0.43 to 5.46, p = 0.27) short-medium term. The combination of the two into an active behavioural intervention may be a good strategy to manage acute WADII.

### Strengths

This study’s strengths are its design and specific focus to acute WADII using a pre-defined protocol and attention to potential sources of bias such as: a minimisation of errors from searching, using two independent reviewers, decreased publication bias through searching both published and unpublished trials, assessment of RoB using two independent reviewers, and data extraction using two reviewers.

### Limitations

This study’s limitations include the small number of trials identified and their high RoB. Furthermore, effectiveness for the outcome of NDI which is a key outcome measure [11, 74] with high validity and reliability [75]could not be calculated in a meta-analysis due to an insufficient number of trials evaluating this outcome.

According to GRADE (the Grading of Recommendations Assessment, Development and Evaluation system),[76] the evidence reviewed in this study is low/very low (low in conservative and active interventions, very low in behavioural intervention). Consequently, an adequately powered, low risk of bias, and well-reported trial to evaluate effectiveness of acute WADII management is warranted to enable confidence in evidence for clinical practitioners, health policy-makers and researchers.

## Conclusions

This rigorous systematic review found that conservative and active interventions may be useful for pain reduction in acute WADII management in the medium-long term. Additionally, improvement of cervical movement in the horizontal plane short term could be promoted by the employment of a conservative intervention. The employment of a behavioural intervention (e.g. act-as-usual, education and self-care including regularly exercise) may be an effective treatment in reducing pain and improving cervical mobility in patients with acute WADII in the short-medium term. Finally, there was no significantly difference between treating in early (<1 week) and late (2 weeks) interventions. The level of evidence from this systematic review is evaluated as low/very low level according to GRADE.

## Supporting Information

S1 Checklist PRISMA Checklist (DOC)
